# A comparative evaluation of CT global noise calculation methods for clinical image quality assessment

**DOI:** 10.1002/acm2.70288

**Published:** 2025-10-14

**Authors:** Charles M Weaver, Gary Ge, Alexander Alsalihi, Jie Zhang

**Affiliations:** ^1^ Division of Diagnostic and Nuclear Medical Physics Department of Radiology University of Kentucky College of Medicine Lexington Kentucky USA

**Keywords:** CT, CMS measure, global noise, image quality assessment

## Abstract

**Background:**

The recently introduced CMS quality measure for computed tomography (CT) requires compliance with two key metrics, radiation dose and image quality, across 18 exam categories. However, the method for calculating global noise (GN) remains undefined, with references to the “Duke method” and “Wisconsin method” as possible options. This lack of clarity raises concerns regarding standardization, compliance, and clinical relevance.

**Purpose:**

To compare GN calculation methods proposed in the Duke and Wisconsin papers and evaluate their variability across clinical CT protocols.

**Methods:**

A retrospective analysis of 719 CT exams was performed across seven protocols, including five abdominal and two chest categories. One protocol (chest PE) included exams reconstructed with both smooth and sharp kernels. Five GN metrics were evaluated: Duke_tissue_mode, Wisconsin_tissue_mean, Wisconsin_tissue_mode, Wisconsin_air_mean, and Wisconsin_air_mode. Statistical differences were assessed using the Friedman test with pairwise Wilcoxon signed‐rank tests, and Pearson correlation matrices were used to evaluate agreement.

**Results:**

Significant differences (*p* < 0.05) were observed across GN metrics in all protocols, with Wisconsin_tissue_mean consistently producing the highest values. Correlation analysis showed strong agreement (*r* > 0.7) for renal stone, chest w/o, and abdomen/pelvis protocols, but weaker correlations for urogram, renal mass, and enterography. Mode‐based metrics showed higher agreement (*r* > 0.9), suggesting dose dependency. In the chest PE protocol, the smooth kernel yielded GN values well below the CMS threshold, while the sharp kernel exceeded the threshold in tissue‐based metrics.

**Conclusions:**

Significant variability across GN metrics highlights the need for a standardized, clinically relevant method. Without clear definitions, the CMS measure's effectiveness in ensuring image quality and dose management may be compromised, an issue also raised by the AAPM‐commissioned panel.

## 1 INTRODUCTION

The Centers for Medicare & Medicaid Services (CMS) have implemented a new computed tomography (CT) quality measure designed to balance radiation dose reduction with the maintenance of diagnostic image quality.^1^ This measure is used to assess the proportion of studies that surpass established thresholds for image noise or radiation dose across various CT examination categories. Integrated into major CMS quality‐based payment programs, this initiative influences reimbursements for hospitals and clinics, with reporting commencing in January 2025 and mandatory compliance slated for 2027.

A critical component of this measure is the calculation of CT global noise (GN), a metric indicative of image quality. However, CMS does not define a specific methodology for this calculation, instead referencing two existing methods developed by Wisconsin and Duke universities, respectively.^2^, ^3^, ^4^ The Wisconsin method employs an automated algorithm to measure noise in CT images, generating a GN level that reflects the standard deviation of Hounsfield units (HU) within specified regions of interest. Conversely, the Duke method focuses on calculating GN magnitude by analyzing soft tissue regions in patient CT images.

The availability of two different methods provides flexibility in reporting, but simultaneously introduces potential variability when assessing CT image quality across institutions. This possibility would lead to GN calculation dependencies and compromise the efficacy of the CMS measure in evaluating institutional image quality. This study aims to assess two existing methods for GN calculations, examining their application across various clinical CT protocols to identify potential discrepancies and implications for maintaining diagnostic integrity while adhering to radiation dose optimization goals.

## 2 METHODS

### 2.1 Patient cohort specifications

CT images were retrospectively collected from seven clinical protocols covering both abdominal and chest imaging. Six protocols were used to assess inter‐method variability, while the seventh, pulmonary embolism (PE), was used to evaluate the effect of reconstruction kernel on GN metrics. As shown in Table 1, the protocols were selected to represent a range of dose categories, consistent with those defined in the UCSF study referenced by the CMS measure.^5^

**TABLE 1:** Selected CT protocols and associated body region and dose categories.

CT protocol	Body region	CMS dose category	CMS threshold (HU)	Number of patients	CTDI_vol_ (mGy) Mean ± std (min–max)	WED (cm) Mean ± std (min–max)
Renal stone	Abdomen	Low	64	100	7.01 **± **3.32 (1.17–26.59)	29.93 **± **3.86 (22.30–41.48)
Abd/Pe; w/o	Abdomen	Routine	29	100	8.45 **± **5.32 (3.07–28.52)	29.82 **± **3.79 (21.86–37.46)
Enterography	Abdomen	Routine	29	105	5.75 **± **3.01 (1.08–18.16)	29.73 **± **3.85 (21.74–40.01)
Urogram	Abdomen	High	29	100	6.82 **± **2.90 (2.66–23.41)	30.28 **± **3.42 (20.62–37.11)
Renal mass w/o	Abdomen	High	29	105	8.81 **± **3.65 (2.43–20.54)	32.74 **± **4.24 (21.52–42.23)
Chest w/o	Chest	Routine	49	104	10.41 **± **6.58 (1.24–26.37)	26.39 **± **3.86 (17.45–39.00)
PE	Chest	High	49	105	11.97 **± **4.74 (1.41–21.96)	26.52 **± **3.67 (18.69–33.80)

Abbreviations: CMS, Centers for Medicare & Medicaid Services; CT, computed tomography; HU, Hounsfield units; PE, pulmonary embolism.

CMS dose thresholds were specified for each protocol.^4^ Each included between 100 and 105 patients, with corresponding mean CTDI_vol_ values and dose ranges reported. For the PE protocol, 45 exams were reconstructed with a soft kernel (Bf37) and 60 with a sharp kernel (Br58), enabling assessment of kernel‐dependent variation in GN measurements. The corresponding CTDI_vol_ values were 11.32  ±  4.43 mGy (range: 3.90–18.58 mGy) for the soft kernel group and 12.46  ±  4.90 mGy (range: 1.41–21.94 mGy) for the sharp kernel group. There was no significant difference observed between the two groups.

Exams were selected based on the following criteria: no anatomical cutoff in the axial middle slice, no visible implanted medical devices, and no arms within the imaging plane. These conditions ensured compatibility with all patient size calculation methods and minimized the impact of high‐attenuation materials. The criteria were applied consistently across all protocols used for inter‐method variability analysis to ensure reliable calculation of both soft tissue and air GN metrics, and to avoid confounding from high‐attenuation artifacts. All scans were acquired on Siemens CT scanners (Siemens Healthineers, Erlangen, Germany), with example acquisition parameters provided in Table 2.

**TABLE 2:** Acquisition parameters for selected CT clinical protocols.

Clinical protocol	Care kV	CareDose4D	Ref. kV	Ref. mAs	Recon kernel	Recon strength	Pitch	Detector configuration (mm)
**Abdomen pelvis w/o**	Yes	Yes	120	150	Br40	2	0.6	192 × 0.6
**Enterography**	Yes	Yes	120	150	Br40	2	0.6	192 × 0.6
**Renal mass w/o**	Yes	Yes	120	150	Br40	2	0.6	192 × 0.6
**Renal stone**	Yes	Yes	120	150	Br40	2	0.6	192 × 0.6
**Urogram**	Yes	Yes	120	150	Br40	2	0.6	192 × 0.6
**Chest w/o**	Yes	Yes	110	55	Br40	3	1.2	192 × 0.6
**Pulmonary embolism**	Yes	Yes	110	125	Bf38/ Br58	3	1.2	192 × 0.6

Abbreviation: CT, computed tomography.

A total of 719 exams were included in the study cohort, with at least 100 exams per CT protocol. A demographic breakdown is provided in Table 3. This retrospective study was conducted in accordance with relevant guidelines and regulations and was approved by the [institution name] Institutional Review Board (IRB).

**TABLE 3:** Patient cohort characteristics.

Characteristics		Number of patients	Percentage of patients (%)
*n*, number of patients		719	NA
			
Gender			
	Male	358	49.8
	Female	361	50.2
			
Age			
	18–29	88	12.2
	30–39	75	10.4
	40–49	93	12.9
	50–59	144	20.0
	60–69	183	25.5
	70–79	105	14.6
	80–89	29	4.0
	90–99	2	0.3

### 2.2 Global noise calculation

Five noise‐related metrics were derived from two established methods: the Duke method and the Wisconsin method. The Wisconsin method proposed performing HU measurements in air or soft tissue and calculating the noise metric using the mean or mode of the measurements, resulting in four possible permutations for GN.^3^ Accordingly, the five GN metrics were named as follows: Duke_tissue_mode, Wisconsin_tissue_mean, Wisconsin_tissue_mode, Wisconsin_air_mean, Wisconsin_air_mode.

Implementation of the GN metrics was done according to the published methodology. Each metric was implemented according to its respective published methodology, as summarized below. Calculations were performed on a slice‐by‐slice basis and averaged across each scan to yield an exam‐level GN value, in accordance with CMS guidance.^1^ All metrics were computed using a custom‐developed program written in Python 3.12 (Python Software Foundation, www.python.org).

#### 2.2.1 Duke method

For each axial slice, a soft tissue segmentation mask was generated using a threshold range from 0 to 100 HU. A 7 mm sliding window was then applied to the unsegmented image to calculate local standard deviations, with each value assigned to the central pixel to form a noise map. This window size was chosen to match the 7 mm region of interest (ROI) used in the Wisconsin method, and the original Duke study demonstrated that window sizes between 6 and 20 mm yielded consistent results.^2^ The soft tissue mask was subsequently applied to the noise map, and the remaining pixel values were compiled into a histogram. The mode (peak value) of this distribution was used as the noise estimate for that slice.^2^

#### 2.2.2 Wisconsin method

The Wisconsin approach consisted of two main steps: noise map generation and image segmentation.

1. Noise map generation

To suppress anatomical structure and isolate random noise, each slice was subtracted from its adjacent neighbor. The resulting image was then divided into a uniform grid of 7 × 7 mm square regions of interest (ROIs).^3^

2. Segmentation

A median filter was applied to the original slice to reduce noise prior to thresholding. Pixels with values between 0 and 100 HU were classified as soft tissue, and those between −1024 and −950 HU were classified as air. Only ROIs entirely contained within the soft tissue or air segmentation masks were included in the analysis.^3^

For each valid ROI, the standard deviation of pixel values was computed. These values were compiled into histograms for both soft tissue and air. From each histogram, two global noise metrics were extracted: the mean, defined as the average standard deviation across ROIs, and the mode, defined as the peak value of the histogram.^3^ This resulted in four GN metrics: Wisconsin_tissue_mean, Wisconsin_tissue_mode, Wisconsin_air_mean, and Wisconsin_air_mode.

**FIGURE 1:**
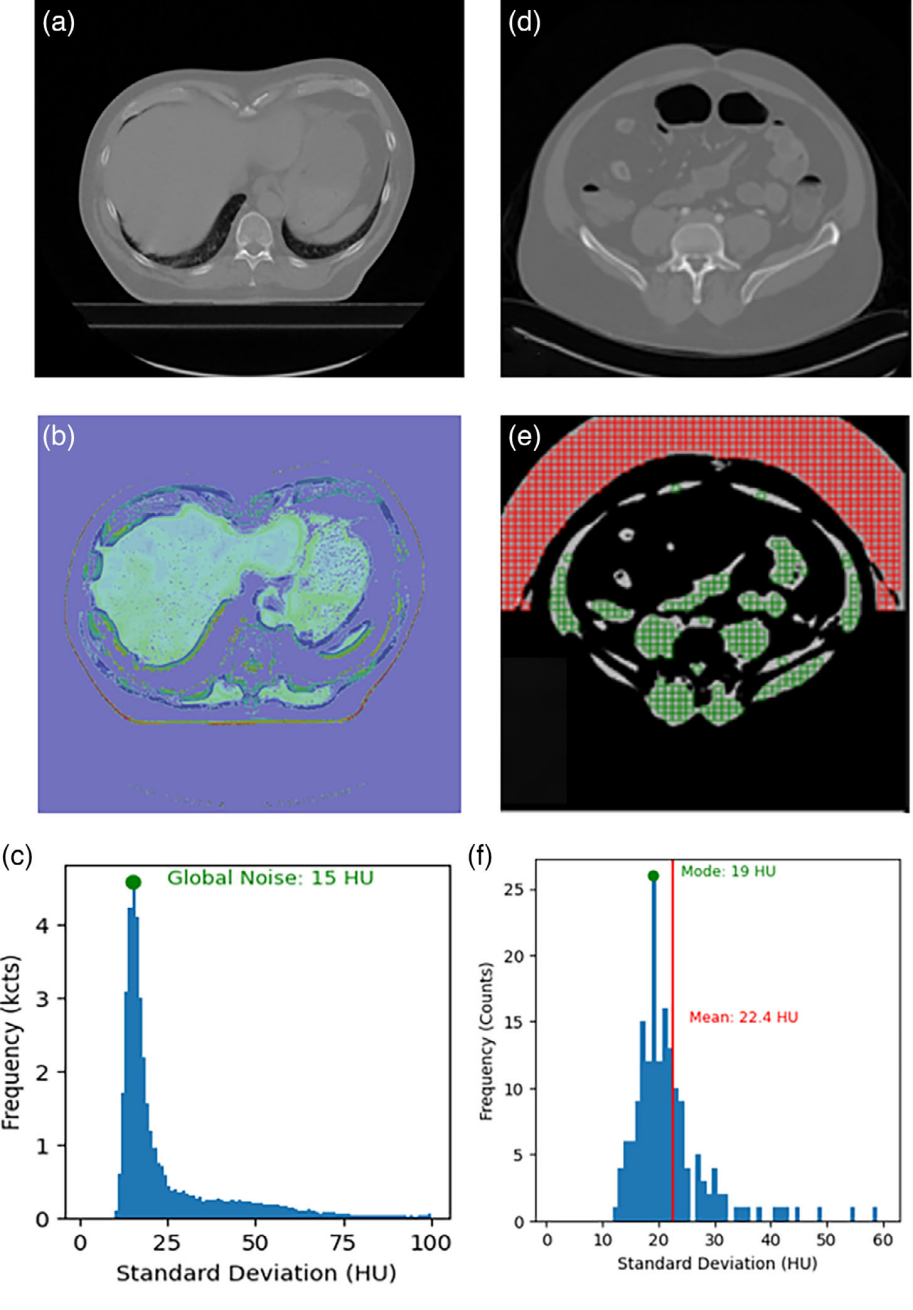
Example of ROI selection and noise metric calculation in a single CT exam. The Duke method is described in parts a–c and the Wisconsin method is described in parts d–f. (a) Axial CT slice from an abdominal scan; (b) Soft tissue segmentation mask (0–100 HU) applied to generate a noise map; (c) Histogram of standard deviations within the masked region. The mode (peak value) of the histogram is used as the global noise value; (d) Axial CT abdominal image; (e) Subtracted image (current slice minus adjacent slice) with overlaid 7 × 7 mm ROI grid. Green ROIs are for tissue noise calculation and red ROIs are for air; (e) Histogram of standard deviations from tissue ROIs. Both the mean and mode are used as global noise values. CT, computed tomography; HU, Hounsfield units; ROI, region of interest.

Figure 1 shows example of ROI selection and noise metric calculation in a CT exam. Figure 1a–c shows the Duke method as described above, while Figure 1d–f described the Wisconsin method for both tissue and air.

### 2.3 Statistical analysis

Two tiers of comparisons were performed: (1) protocol‐level evaluations of GN metrics to assess differences and inter‐metric correlations within each CT protocol, and (2) patient‐level comparisons of soft tissue GN metrics to determine whether different approaches yield significantly different exam‐level values. Analyses were conducted using Python (version 3.12.7) and SciPy (version 1.13.1).

For each CT protocol, Friedman tests were used to assess overall differences among GN metrics. When significant, pairwise Wilcoxon signed‐rank tests with Holm–Bonferroni correction were applied to identify specific metric pairs with significant differences.

Pearson correlation coefficients were calculated for all pairwise metric combinations within each protocol. These correlations were visualized as heatmaps to illustrate the strength and pattern of relationships among GN metrics across CT protocols.

At the patient level, paired permutation tests were conducted among the three soft tissue GN metrics to evaluate whether they produced statistically different exam‐level values. Each exam was treated as a matched set of slice‐level measurements, with linkage preserved during permutation to account for within‐scan dependencies, ensuring valid inference for scan‐averaged comparisons.

## 3 RESULTS

**FIGURE 2:**
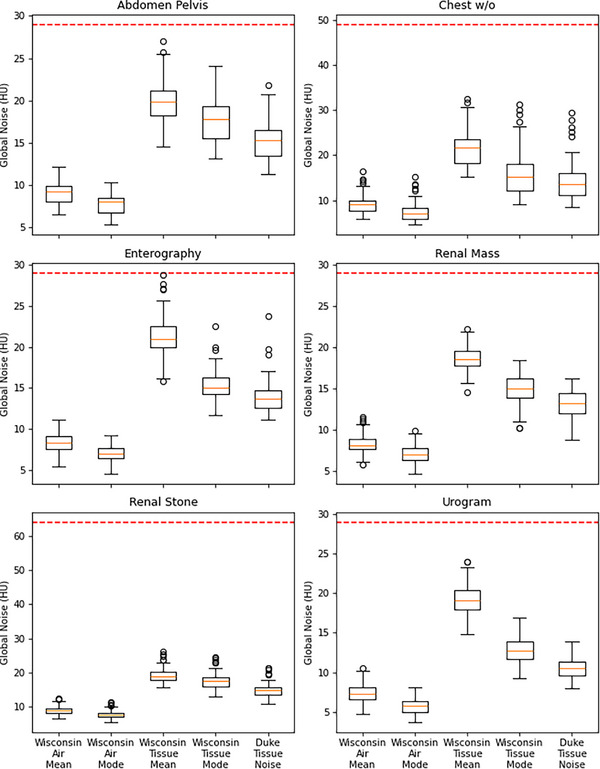
Box plots of GN metrics for six protocols, highlighting differences between Wisconsin_tissue_mean, Wisconsin_tissue_mode, and Duke_tissue_mode for renal mass, chest w/o, abdomen/pelvis, enterography, renal stone, and urogram protocols. The air‐based metrics generate lower GN values than tissue‐based metrics, and the Wisconsin_tissue_mean metric is consistently the highest. All metrics are significantly different from each other (*p* < 0.05), indicating that metric selection will impact an institution's reported values. The dotted line indicates CMS noise threshold. CMS, Centers for Medicare & Medicaid Services; GN, gobal noise.

Table 4 shows GN values across seven CT protocols using five different GN metrics. Wisconsin_tissue_mean generates the largest GN values and Wisconsin_air_mode generates the smallest GN values, consistently. Duke_tissue_mode generates the lowest GN value among the tissue‐based metrics for all protocols except chest PE. Friedman tests and subsequent pairwise analysis demonstrate significant differences (*p* < 0.05) between GN metrics in all protocols (Figure 2). Paired permutation tests reveal significant differences in tissue noise metrics for nearly all patients, with only five out of 719 cases showing no significant variation. Larger variability is noted in the urogram protocol compared to other exam types, indicating potential limitations in the reliability of certain noise metrics for high‐dose studies.

**TABLE 4:** A summary of global noise (GN) metrics across seven CT protocols for five GN metrics. The unit is in Hounsfield Units (HU).

		Renal stone	Abdomen Pelvis w/o	Enterography	Urogram	Renal mass w/o	Chest w/o	PE
Duke_tissue_mode	Average	14.95	15.16	13.95	10.52	13.20	14.66	51.51
	Std	2.07	2.35	1.89	1.18	1.63	6.63	34.43
	Range	10.84–21.35	11.22–21.85	11.15–23.71	7.97–13.90	8.76–16.28	8.50–37.30	9.14–96.14
Wisconsin_tissue_mean	Average	19.21	19.92	21.25	19.25	18.41	21.94	51.21
	Std	2.09	2.68	2.18	1.84	1.36	7.01	32.17
	Range	15.61–26.07	14.55–27.04	15.81–28.78	14.84–23.98	14.61–22.25	15.14–82.21	11.73–94.85
Wisconsin_tissue_mode	Average	17.55	17.69	15.38	12.73	14.92	16.23	47.02
	Std	2.34	2.78	1.79	1.49	1.79	7.33	32.16
	Range	12.96–24.66	13.11–24.04	11.65–22.54	9.22–16.86	10.22–18.50	9.04–74.86	7.44–91.97
Wisconsin_air_mean	Average	9.01	9.13	8.34	7.41	8.42	9.44	21.75
	Std	1.25	1.30	1.24	1.18	1.18	3.30	13.30
	Range	6.51–12.36	6.55–12.14	5.44–11.19	4.68–10.58	5.82–11.64	5.87–36.18	4.75–39.36
Wisconsin_air_mode	Average	7.78	7.75	6.99	5.68	7.08	7.58	19.62
	Std	1.10	1.16	0.94	0.86	1.05	1.69	12.32
	Range	5.49–11.53	5.29–10.29	4.59–9.24	3.68–8.04	4.75–9.87	4.61–33.65	3.82–37.05

Correlation analysis indicates strong agreement (*r* > 0.7) among the five global noise metrics for renal stone, chest w/o, and abdomen/pelvis protocols. Weaker correlations are observed for urogram, renal mass, and enterography protocols. The strength of these correlations appear to be dose‐dependent, with mode‐based global noise metrics exhibiting stronger agreement, often exceeding *r* > 0.9.

**FIGURE 3:**
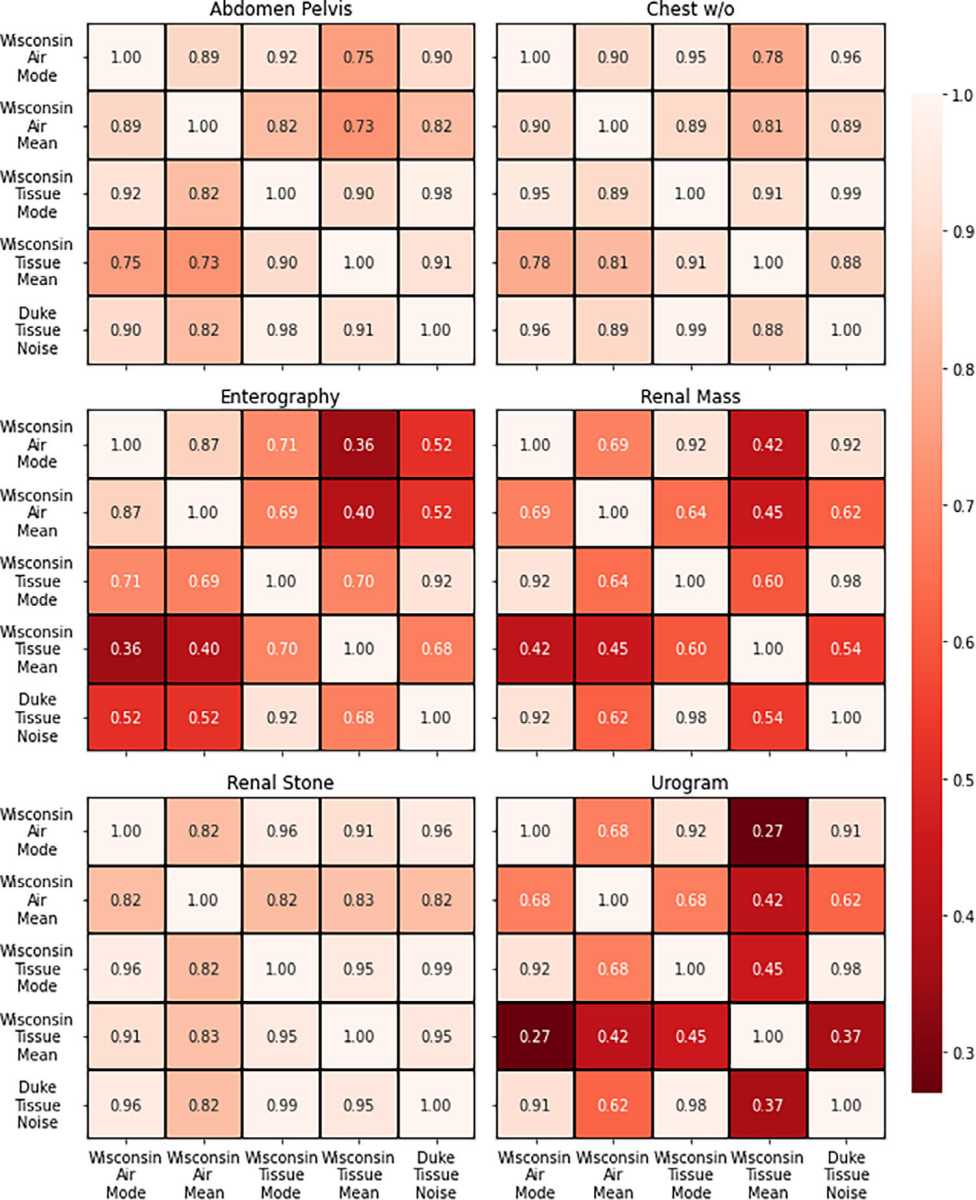
Heatmaps showing correlation strengths for global noise metrics per protocol. Abdomen/pelvis: 8/10 show very strong correlations (*r* ≥ 0.8), 2/10 strong (0.6 ≤ *r* < 0.8); chest w/o: 9/10 very strong, 1/10 strong; renal stone: 10/10 very strong; urogram: 3/10 very strong, 3/10 strong, 2/10 moderate (0.4 ≤ *r* < 0.6), 2/10 weak (0.2 ≤ *r* < 0.4); enterography: 2/10 very strong, 4/10 strong, 3/10 moderate, 1/10 weak; renal mass: 3/10 very strong, 4/10 strong, 3/10 moderate.

Correlation matrices (Figure 3) illustrates that the abdomen/pelvis protocol is consistent, with eight out of ten comparisons showing very strong correlations (*r* ≥ 0.8) and the remaining two classified as strong (0.6 ≤ *r* < 0.8). Similarly, the chest w/o protocol exhibits nine very strong correlations (*r* ≥ 0.8) and one strong correlation (0.6 ≤ *r* < 0.8). The renal stone protocol shows the highest level of agreement, with all ten comparisons classified as very strong (*r* ≥ 0.8). In contrast, the urogram, renal mass, and enterography protocols display weak correlations, highlighting inconsistencies in GN metric performance across different CT exam types.

**FIGURE 4:**
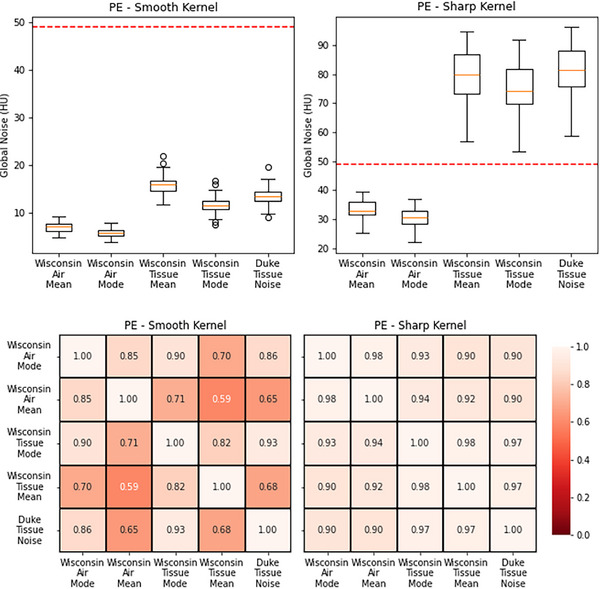
Boxplots and correlation matrices for the chest PE protocol. The patient cohort was evenly split between a smooth and sharp reconstruction kernel. The global noise values for the sharp kernel exceed the CMS threshold in the tissue noise metrics. The correlation matrix for the smooth kernel shows low correlations similar to the urogram protocol, where the sharp kernel has high correlations similar to the chest w/o protocol. The dotted line indicates CMS noise threshold. CMS, Centers for Medicare & Medicaid Services.

The results for the chest PE protocol are shown in Figure 4. The smooth reconstruction kernel has GN metric values that are well below the CMS‐defined threshold, where the sharp reconstruction kernel has values that are above the threshold in the tissue noise metrics. The correlation matrix for the smooth reconstruction kernel shows similar behavior to the urogram (high dose) and renal mass (high dose) CT protocols, whereas the sharp kernel is similar to the renal stone (low dose), chest w/o (routine dose) and abdomen/pelvis (routine dose) protocols.

## 4 DISCUSSION

Significant variations are observed across the five GN metrics when applied to different CT protocols. Each metric also behaves differently across protocols, which may be due to differences in the examinations and scan parameters. While some variation is expected, GN values vary substantially based on the chosen metric, with air‐based metrics consistently yielding lower values. Even among similar metric types, notable discrepancies are present.

GN is highly sensitive to variables such as slice thickness, iterative reconstruction (IR) strength, and kernel selection. However, standardizing CT protocols is nearly impossible due to institutional dependencies (e.g., radiologist preferences, local patient populations, vendor selections) and both inter‐ and intra‐vendor variability. For example, the AAPM reference lung cancer screening protocols show variation in scan parameters between vendors as well as different models made by the same vendor. Modern image reconstruction methods (e.g., IR, deep‐learning) add additional complexity to this through different reconstruction strengths.

Although CMS addresses slice thickness by standardizing to a 3 mm reference, it does not account for other scan parameters. For example, as shown in Figure 4, chest PE protocols typically use sharp kernels for clinical diagnosis, but a smooth reconstruction that is part of the protocol would result in lower image noise and would influence GN values, potentially misrepresenting true clinical image quality and undermining the intent of the CMS measure. Furthermore, focusing only on global noise amplitude ignores noise texture, which significantly impacts perceived image quality and diagnostic confidence.^6^

It is worth noting that the current CMS measure lacks clarity regarding both the choice of GN metric and the method for defining GN thresholds. This ambiguity may enable institutions to report artificially low GN values that fall below thresholds, irrespective of actual image quality. However, the National Quality Forum (NQF) submission for the CMS measure states^4^ “Noise as defined in this measure is calculated on every CT image within a scan (a single irradiating event), and the global noise value for each scan is the mean value across all images.” For CT exams that have multiple scans, the exam is assigned the “best” global noise value across all scans, that is, the highest quality scan. Based on this statement, the NQF submission implies that all reconstructed image series from an irradiating event are included in GN calculations, regardless of their clinical relevance. This approach introduces additional variability and creates opportunities for gaming, particularly when non‐diagnostic series are present.

The selection of the GN threshold warrants reconsideration. Although all protocols produced global noise values below the CMS‐defined thresholds, the renal stone and chest w/o protocols demonstrated substantially lower values, with no calculation method yielding a median value exceeding 50% of the CMS threshold. Threshold selection should be guided by the image quality metric used, where tissue‐based metrics require higher thresholds, and air‐based metrics require lower ones. However, variability in acquisition techniques and the lack of standardized reconstruction parameters or noise metrics make establishing meaningful thresholds a significant challenge.

Our findings align with concerns raised by the AAPM‐commissioned panel evaluating the CMS measure.^7^ Without a standardized GN metric and reporting framework, the measure may fail to achieve its intended goals of promoting consistent CT image quality and dose optimization. The choice of GN metric directly impacts compliance and inter‐institutional comparability. A clearly defined, clinically meaningful, and universally adopted metric is essential to ensure: consistent application of regulatory standards, reliable image quality assessment, and fair comparison of dose optimization efforts across institutions.

This study has a few limitations. We did not evaluate all 18 CMS CT Dose and Image Quality categories, but this will not impact the validity of our main conclusions. Due to the retrospective nature of the study, we also did not investigate the impact of slice thicknesses other than 3 mm or IR reconstruction strength. However, the significant differences observed between two reconstruction kernels in the PE protocol suggest that similar variability would be seen with other acquisition parameters. In addition, the selection criteria for the patient cohort ensured that both the air‐ and tissue‐based metrics were able to be calculated, but it is possible that CT protocols could acquire images without air (e.g., small FOV cardiac studies, large patient habitus). How these cases would be handled does not appear to be clarified in the measure document.

In conclusion, this study provides a critical assessment of two methods for calculating CT global noise referenced in the upcoming CMS measure. By comparing five GN metrics across multiple clinical protocols, we identify substantial metric‐dependent variability. Together with variations in size‐adjusted dose estimation methods,^8^ these findings underscore the need for a standardized, clinically appropriate metrics to ensure consistency in evaluating image quality and radiation dose, as well as compliance with regulatory standards.

## AUTHOR CONTRIBUTIONS

Charles M. Weaver developed the software used in the study and performed the statistical analysis. Gary Ge drafted the manuscript, supervised data collection, and contributed to data analysis. Alexander Alsalihi retrieved all CT images for analysis. Jie Zhang contributed to clinical data acquisition and analysis, provided critical revisions to the manuscript, and supervised the project.

## CONFLICT OF INTEREST STATEMENT

The authors have no conflicts of interest to disclose

## ETHICS STATEMENT

IRB approval obtained from the University of Kentucky.
